# [^18^F]-BMS-747158-02PET imaging for evaluating hepatic mitochondrial complex 1dysfunction in a mouse model of non-alcoholic fatty liver disease

**DOI:** 10.1186/s13550-017-0345-5

**Published:** 2017-12-06

**Authors:** Takemi Rokugawa, Sotaro Momosaki, Miwa Ito, Hitoshi Iimori, Yuki Kato, Kohji Abe

**Affiliations:** 1 Translational Research Unit, Biomarker R&D Department, Shionogi Co., Ltd, 3-1-1, Futaba-cho, Toyonaka, Osaka 561-0825 Japan; 2 Department of Applied Chemistry & Analysis, Research Laboratory for Development, Shionogi & Co., Ltd, Osaka, Japan; 3 Department of Drug Safety Evaluation, Research Laboratory for Development, Shionogi Co., Ltd, Osaka, Japan; 4 Molecular Imaging in Medicine, Osaka University Graduate School of Medicine, Osaka, Japan

**Keywords:** [^18^F]-BMS747158-02, Non-alcoholic fatty liver disease, Non-alcoholic steatohepatitis, Mitochondrial complex-1, Methionine- and choline-deficient diet, Positron emission tomography

## Abstract

**Background:**

Mitochondrial dysfunction is one of the main causes of non-alcohol fatty liver disease (NAFLD). [^18^F]-BMS-747158-02 (^18^F-BMS) which was originally developed as a myocardial perfusion imaging agent was reported to bind mitochondrial complex-1 (MC-1). The aim of this study was to investigate the potential use of ^18^F-BMS for evaluating hepatic MC-1 activity in mice fed a methionine- and choline-deficient (MCD) diet.

Male C57BL/6J mice were fed a MCD diet for up to 2 weeks. PET scans with ^18^F-BMS were performed after 1 and 2 weeks of the MCD diet. ^18^F-BMS was intravenously injected into mice, and the uptake (standardized uptake value (SUV)) in the liver was determined. The binding specificity for MC-1 was assessed by pre-administration of rotenone, a specific MC-1 inhibitor. Hepatic MC-1 activity was measured using liver homogenates generated after each positron emission tomography (PET) scan. Blood biochemistry and histopathology were also assessed.

**Results:**

In control mice, hepatic ^18^F-BMS uptake was significantly inhibited by the pre-injection of rotenone. The uptake of ^18^F-BMS was significantly decreased after 2 weeks of the MCD diet. The SUV at 30–60 min was well correlated with hepatic MC-1 activity (*r* = 0.73, *p* < 0.05). Increases in plasma ALT and AST levels were also noted at 1 and 2 weeks. Mild hepatic steatosis with or without minimal inflammation was histopathologically observed at 1 and 2 weeks in mice liver on the MCD diet. However, inflammation was observed only at 2 weeks in mice on the MCD diet.

**Conclusions:**

The present study demonstrated that ^18^F-BMS is a potential PET probe for quantitative imaging of hepatic MC-1 activity and its mitochondrial dysfunction induced by steatosis and inflammation, such as in NAFLD.

## Background

Non-alcoholic fatty liver disease (NAFLD) is one of the most common forms of chronic liver disease in patients without a history of alcoholic abuse [1]. NAFLD encompasses a wide spectrum of conditions ranging from simple steatosis to non-alcoholic steatohepatitis (NASH), which progress to fibrosis in 30–40% of patients and to cirrhosis in 10–15% of patients [2]. Despite the poor prognosis, diagnosis of NAFLD including NASH is difficult because liver biopsy, which is an invasive method, is the gold standard to identify steatohepatitis [3]. This has both made it difficult to diagnose NAFLD and to identify the mechanism of progression from simple steatosis to NASH, exacerbated by its complicated pathogenesis. One of the key factors in the acceleration of progression from simple steatosis to NASH is the formation of reactive oxygen species (ROS) [4, 5]. ROS directly damage respiratory chain polypeptides and oxidize the unsaturated lipids of cytoplasmic hepatic fat deposits to cause lipid peroxidation. Both ROS and lipid peroxidation products attack mitochondrial DNA [6]. Oxidative mitochondrial DNA lesions and mitochondrial DNA depletion may cause mitochondrial dysfunction including in energy metabolism. Mitochondrial alterations have been reported in patients with NASH [7] and are associated with impairment of hepatic ATP synthesis [8]. Therefore, mitochondrial dysfunction is a key factor in the progression from steatosis to steatohepatitis. In rats fed a choline-deficient diet, which is well known as a fatty liver disease model, mitochondrial membrane potential was decreased with no inflammation [9]. Thus, mitochondrial dysfunction might occur in the early phase of NAFLD.

^99m^Tc-MIBI is a single photon emission computed tomography (SPECT) probe which accumulates mitochondrial membrane potential [10, 11]. Previously, we reported that SPECT using ^99m^Tc-MIBI could detect mitochondrial dysfunction in mice fed a methionine- and choline-deficient (MCD) diet, a commonly used NAFLD model [12]. In clinical studies, it has been reported that ^99m^Tc-MIBI uptake correlated with NAFLD activity score [13]. Therefore, evaluation of mitochondrial dysfunction enables detection of the early phase of NAFLD. It is known that positron emission tomography (PET) has a higher resolution and better quantitation than SPECT [14]. [^18^F]-BMS-747158-02 (^18^F-BMS) was originally developed as a myocardial perfusion imaging agent [15, 16]. The uptake of ^18^F-BMS was reported to depend on mitochondrial complex-1 (MC-1) activity, which is the first component of the mitochondrial respiratory electron transport chain. Recently, Ohba et al. reported that [^18^F]-BCPP-BF, which was used as a PET probe for MC-1 like ^18^F-BMS, detected liver dysfunction in an acetaminophen-treated rat hepatic injury model [17]. These findings suggest that ^18^F-BMS might also have the potential to detect mitochondrial dysfunction as a cause of NAFLD. In our previous study, ^99m^Tc-MIBI could detect the mitochondrial membrane dysfunction in 2 weeks in MCD diet-fed mice. In addition, significant loss of body weight was observed in 3 weeks in MCD diet-fed mice.

In the present study, we evaluated the ability of ^18^F-BMS to act as a PET ligand for the detection of hepatic MC-1 activity using mice fed a MCD diet to clarify the mitochondrial dysfunction in the early phase of NAFLD.

## Methods

### Animals and experimental design

Male C57BL/6J mice, aged 8 weeks old, were purchased from CLEA Japan (Shizuoka, Japan). The mice were studied after 1 or 2 weeks on a MCD diet (Dyets, Bethlehem, PA, USA) or a normal diet (control group). They were allowed free access to chow and tap water and were housed in a temperature-controlled room maintained on a 12-h light/dark cycle with lights on at 8:00 am. The experimental protocols were reviewed and approved by the Institutional Animal Care and Use Committee of Shionogi Research Laboratories and Osaka University Graduate School of Medicine.

### Radiopharmaceutical preparation

**Fig. 1:**
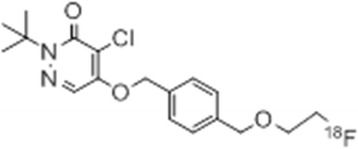
Structure of ^18^F-BMS-747158-02

The chemical structure of ^18^F-BMS is shown in Fig. 1. ^18^F-BMS was synthesized as reported previously [18]. Briefly, after the solvent of the 22–30 GBq ^18^F-F^−^ eluate was evaporated under a stream of nitrogen, approximately 5.0 mg of tosylate precursor dissolved in anhydrous acetonitrile was added. After 20 min incubation at 110 °C and then cooling to room temperature, the reaction mixture was injected onto a semi-preparative HPLC column (COSMOSIL 5C18 MS-II 10 × 250 mm Nacalai Tesque Kyoto, Japan), mobile phase 30 mM ammonium acetate and acetonitrile (3:2), and flow rate 5.0 mL/min for purification. The desired radioactive fraction was collected in a round-bottom flask, and the solvent was removed in vacuo and the residue was dissolved in 1.5 mL of saline:EtOH:Tween80 (4:0.3:0.05) solution. The radiochemical yield was 20–30% (non-decay-corrected). Radiochemical purity and specific activity were 98.7% ± 1.2% and 162.9 ± 57.8 GBq/μmol, respectively, with a radiosynthesis and purification time of 60 min.

### PET scan

PET scan and X-ray CT imaging were performed with a Pre-Clinical Imaging System Triumph LbPET12/CT (TriFoil Imaging Inc., Chatsworth, CA, USA). Control mice, rotenone-injected mice, and mice fed a MCD diet for 1 and 2 weeks were anesthetized with 2% isoflurane. In the group of rotenone, control mice were injected with rotenone (1 mg/kg, i.p.) 20 min before ^18^F-BMS injection. Six to seven mice per group were used for PET imaging. Under isoflurane anesthesia, a venous catheter was introduced through the tail vein and used for the administration of ^18^F-BMS. Approximately 10–20 MBq ^18^F-BMS was continuously injected (0.2 mL/30 s) into the tail vein. PET scans were started immediately after ^18^F-BMS injection was started. Dynamic data acquisition was performed for 60 min. After the PET scans, CT scans were performed to acquire anatomical information and to obtain the data for attenuation collection of PET images. The CT images were reconstructed using the filtered back-projection method (512 slices), and acquired PET images were reconstructed by the 3D-MLEM method with CT-based attenuation collection. Dynamic images (6 × 10 s, 4 × 1 min, 11 × 5 min) for time activity curve (TAC) as well as summation images (30–60 min) were reconstructed. CT and PET images were automatically fused by AMIDE 0.9.2 software. Two regions-of-interest (ROI) were put on the liver tissue, excluding the portal area. Liver uptake of radioactivity was decay-corrected to the injection time and was expressed as the standardized uptake value (SUV), where SUV = tissue radioactivity concentration (MBq/cm^3^)/injected radioactivity (MBq) × body weight (g). After the PET/CT scan, each mouse was euthanized and the liver collected. Livers were immediately frozen on dry ice and stored at − 80 °C until the MC-1 complex activity measurement or processed for histology.

### Measurement of hepatic MC-1 activity

Hepatic MC-1 activity was measured on liver homogenates using the Complex 1 Enzyme Activity Dipstick Assay Kit (#MS130; MitoSciences, Eugene, OR, USA). Briefly, MC-1 was immunocaptured and immunoprecipitated in active form on the dipstick. Then, the dipstick was immersed in MC-1 activity buffer solution containing NADH as a substrate and nitrotetrazolium blue as the electron acceptor. Immunocaptured MC-1 oxidized NADH and the resulting H^+^ reduced nitrotetrazolium blue to form a blue-purple precipitate. The signal intensity of this precipitate corresponded to the level of MC-1 enzyme activity in the sample. The total protein concentration of the liver homogenate samples were measured using a Protein Assay Kit (Bio-Rad, Hercules, CA, USA).

### Blood biochemistry and histopathology

Mice were euthanized by exsanguination under isoflurane anesthesia. Plasma was collected and assayed for the content of alanine aminotransferase (ALT), aspartate aminotransferase (AST), triglyceride (TG), total cholesterol (TC), and high-density lipoprotein cholesterol (HDLC). The right hepatic lobes were fixed in 10% formalin and sectioned, and the 4 μm sections were stained with hematoxylin and eosin (H&E). Steatosis and inflammation in the liver were comprehensively assessed by two pathologists based on severity and size of the lesion. Histopathological scores ranged from 0 to 4 (normal 0, minimal 1, mild 2, moderate 3, marked 4).

### Statistical analysis

Quantitative data are expressed as the means ± SEM. Means were compared using Dunnett’s test. *p* values < 0.05 were considered to indicate statistically significant differences. The Pearson product-moment correlation coefficient was used to evaluate the relationship between SUV of ^18^F-BMS in the liver and the hepatic MC-1 activity.

## Results

### Physiological characteristics and hepatic pathology

**Fig. 2:**
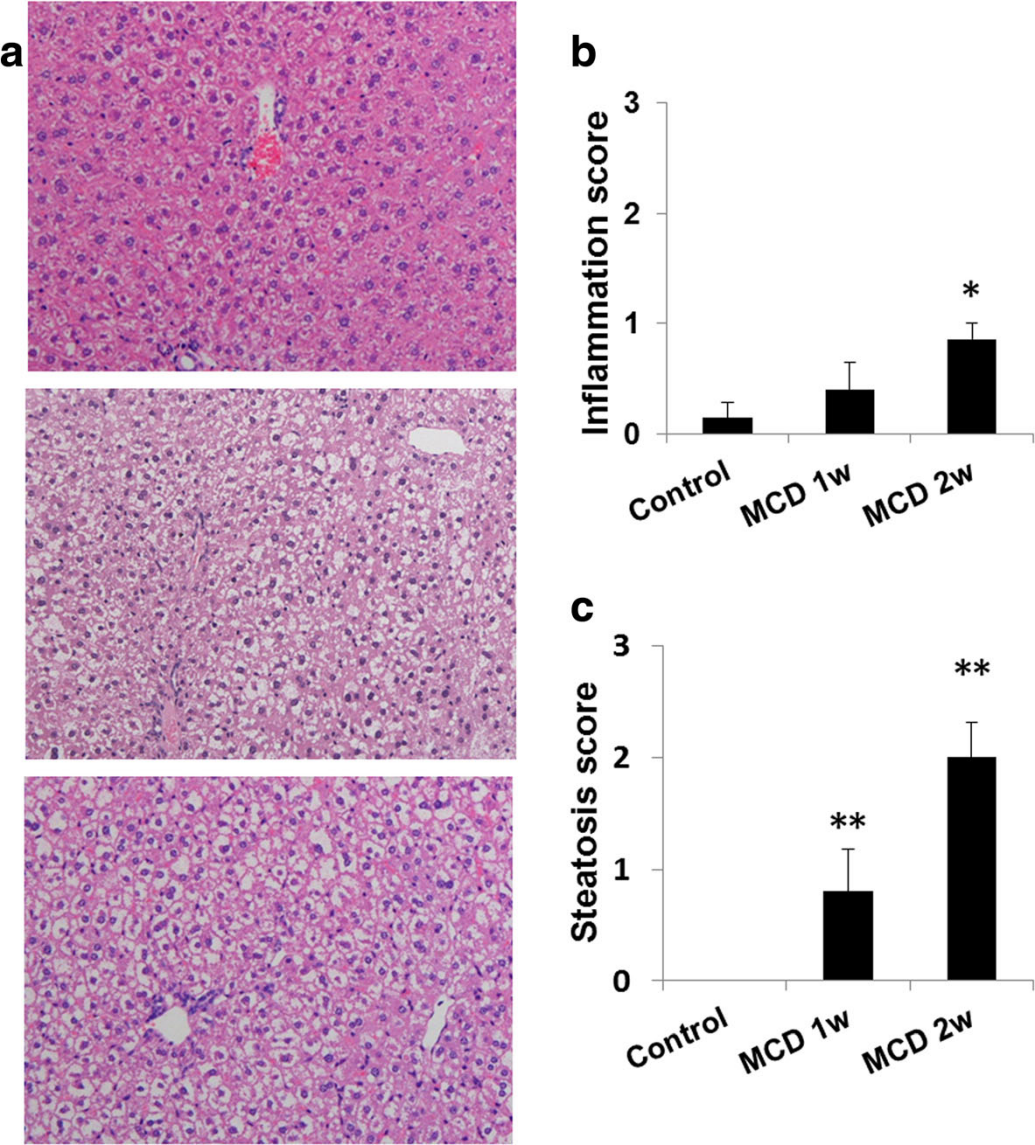
Hepatic histopathology in mice fed a normal or methionine- and choline-deficient (MCD) diet for 1 or 2 weeks. **a** Representative photomicrographs of the liver. Histopathological scores ranged from 0 to 4 (normal 0, minimal 1, mild 2, moderate 3, marked 4). Upper, control diet; middle, MCD diet 1 week; lower, MCD diet 2 weeks. **b** Inflammation score in the liver of treated mice. **c** Steatosis score in the liver of treated mice. Data are expressed as the mean ± SEM, *n* = 5–7 mice per group. *p < 0.05 and **p < 0.01 compared with the control mice. Hematoxylin and eosin (H&E), × 200

Plasma ALT and AST levels were significantly elevated after 1 and 2 weeks of the MCD diet as compared with those of the control mice (*p* < 0.05) (Table 1). In mice fed the MCD diet for 1 week, weak steatosis, but no inflammation, was observed in the liver. In mice fed a MCD diet for 2 weeks, mild steatosis and minimal inflammation were observed (Fig. 2, Table 1).

**Table 1:** Plasma parameters in mice fed control or methionine- and choline-deficient (MCD) diet for 1 and 2 weeks

Parameter	Control	MCD 1 week	MCD 2 weeks
TC (mg/dL)	91.1 ± 2.60	66.5** ± 3.85	52.3** ± 2.23
TG (mg/dL)	99.9 ± 15.6	31.6** ± 2.67	7.43** ± 1.71
AST (IU/L)	59.4 ± 18.6	367** ± 45.9	393** ± 108
ALT (IU/L)	25.1 ± 7.16	405** ± 67.1	256** ± 83.0
HDLC (mg/dL)	50.6 ± 1.61	36.1** ± 3.59	27.9** ± 2.08

Data are expressed as the mean ± SEM of six mice per group. Statistical differences were assessed using Dunnett’s test

*TC* total cholesterol, *TG* triglyceride, *AST* aspartate transaminase, *ALT* alanine aminotransferase, *HDLC* high-density lipoprotein cholesterol

**p < 0.01 compared with the control mice

### Mitochondrial complex 1 activity

**Fig. 3:**
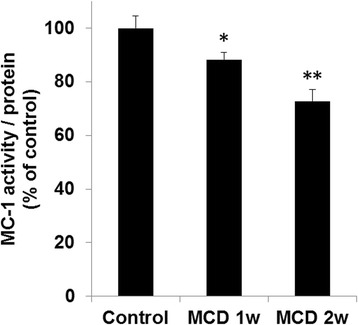
Hepatic mitochondrial complex-1 (MC-1) activities in mice fed a control or methionine- and choline-deficient (MCD) diet. Data are expressed as the mean ± SEM, *n* = 6–7 mice per group. Statistical differences were assessed using Dunnett’s test. *p < 0.05 and **p < 0.01 compared with the control mice

Hepatic MC-1 activity was evaluated after PET imaging. MC-1 activity was decreased by 12% (*p* < 0.05) in mice fed a MCD diet for 1 week and by 27% (*p* < 0.01) in mice fed a MCD diet for 2 weeks compared with the control group (Fig. 3).

### PET scans

**Fig. 4:**
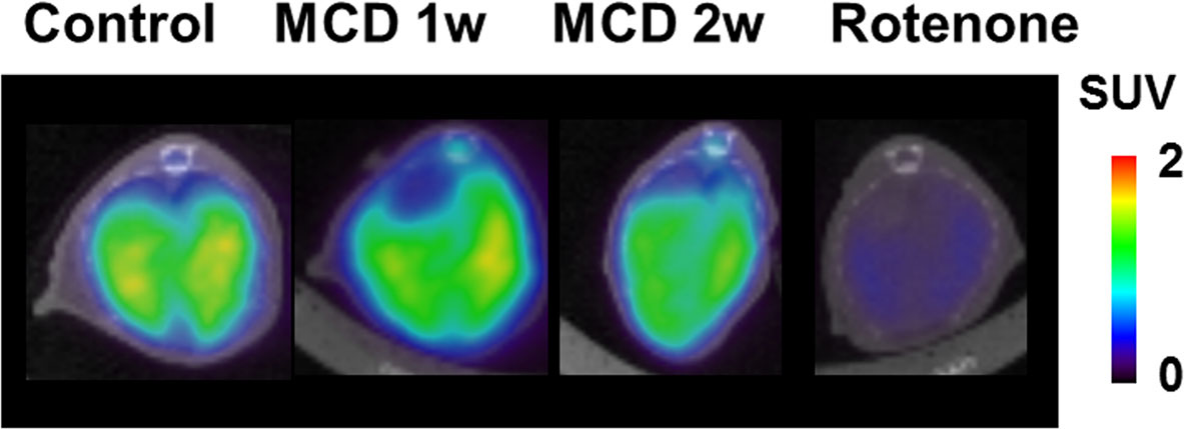
Representative PET/CT fusion images in the livers of mice fed a control or methionine- and choline-deficient (MCD) diet. Inhibition studies were performed by pre-injection of rotenone (1 mg/kg) into control mice 20 min before ^18^F-BMS infusion

**Fig. 5:**
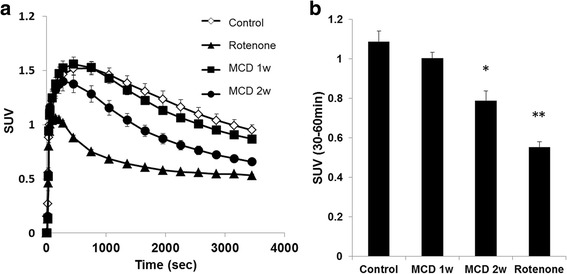
Hepatic radioactivity after ^18^F-BMS injection in mice fed a control or methionine- and choline-deficient (MCD) diet. Rotenone (1 mg/kg, i.p.) were injected into control mice 20 min before ^18^F-BMS injection. **a** Time-activity curve of ^18^F-BMS in control mice, MCD diet mice, and rotenone pre-injection mice. **b** SUV in control mice, MCD diet mice, and rotenone pre-injection mice. *n* = 6–7 mice per group. *p < 0.05 and **p < 0.01 compared with the control mice

^18^F-BMS PET scans were performed on control mice, rotenone-injected mice, and mice fed a MCD diet. Typical summed axial slice PET images and time activity curves (TACs) of the liver are shown in Figs. 4 and 5a. ^18^F-BMS was taken up rapidly by the liver and slowly washed out (Fig. 5a). SUV at 30–60 min revealed that ^18^F-BMS uptake was 50% lower in rotenone-injected mice than control mice (1.09 vs 0.55, *p* < 0.01) (Fig. 5b). As shown in Fig. 5, a prolonged MCD diet accelerated ^18^F-BMS clearance from the liver. In mice fed a MCD diet for 1 week, uptake of ^18^F-BMS was slightly decreased, but there was no significant difference compared with control mice (1.09 vs 1.00, *p* > 0.05) (Fig. 5). In contrast, ^18^F-BMS uptake of mice fed a MCD diet for 2 weeks was decreased by 27% as compared with control mice (1.09 vs 0.79, *p* < 0.05).

**Fig. 6:**
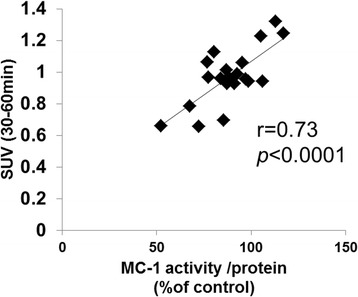
Correlation between hepatic ^18^F-BMS uptake and mitochondrial complex 1 (MC-1) activity in mice fed control and MCD diet. Correlation analysis using the Pearson product-moment correlation

Correlation analysis between ^18^F-BMS uptake and MC-1 activity indicated a positive correlation (*r* = 0.73, *p* < 0.0001) (Fig. 6).

## Discussion

In the present study, ^18^F-BMS was used to evaluate hepatic MC-1 activity in mice fed MCD diet as a model of NAFLD. ^18^F-BMS has been investigated as a PET myocardial perfusion imaging agent for both clinical and non-clinical use [19, 20]. ^18^F-BMS was reported to bind tightly to cellular MC-1, which is the first component of the four electron transport complexes in the inner mitochondrial membrane [16]. ^18^F-BMS is selectively taken up into the heart because of the high density of mitochondria in the cardiac muscle. There are some reports that uptake of MC-1 probes, including ^18^F-BMS, was inhibited by pre-injection of rotenone, a MC-1 inhibitor not only in the heart but also in the brain [21, 22]. In the present study, hepatic uptake of ^18^F-BMS was also reduced by pre-injection of rotenone, indicating that ^18^F-BMS is bound to MC-1 in the liver. Furthermore, the most interesting finding of the present study was that hepatic MC-1 activity was correlated with hepatic ^18^F-BMS uptake. Thus, these results indicate that hepatic uptake of ^18^F-BMS also depends on MC-1 activity. In the present study, a MC-1 immunocaptured dipstick assay kit, which measured MC-1-specific NADH oxidase activity was used. This method has been used in several tissues, including mouse liver, and was previously used to detect a tetracycline-induced decrease in hepatic MC-1 activity [23]. Notably, MC-1 activity was also significantly decreased at 2 weeks in mice fed a MCD diet. In a rat study, 11 weeks of a MCD diet decreased MC-1 activity over 70% [24]. It was also reported that a prolonged MCD diet progressed NAFLD pathology [24, 25]. In our previous study, NAFLD severity increased with duration of MCD diet [25]. Although after 2 weeks of the MCD diet no fibrosis was observed, 4 weeks of a MCD diet induced weak fibrosis and liver fibrosis was clearly observed at 6 weeks [25]. In the present study, 1 and 2 weeks of a MCD diet revealed weak or mild steatosis and weak inflammation in mice. Therefore, up to 2 weeks of a MCD diet represents a model for early-stage NAFLD. Multiple studies support the observation that mitochondrial dysfunction is involved in the development of NASH [8, 26, 27]. Mitochondria generate ROS which damage the mitochondrial respiratory complex, decrease mitochondrial membrane potential, and cause ATP depletion [27]. In the setting of NAFLD, there are reports regarding mitochondrial respiratory chain enzymes in NASH patients. Perez et al. reported a lower activity of the five mitochondrial respiratory complexes in patients with NASH [28]. Thus, our study indicated that ^18^F-BMS might be useful as a high-resolution imaging method for the diagnosis of patients with NAFLD.

In an in vivo microscopic study, blood perfusion of the liver of mice fed MCD diet for 3 and 5 weeks was decreased by 13 and 19% respectively [29]. There was no study to evaluate blood perfusion of the liver of mice fed a MCD diet for 1 or 2 weeks. In our previous dynamic enhanced MRI study, *T*_max_ and *T*_1/2_ after injection of gadolinium-ethoxybenzyl-diethylenetriamine penta-acetic acid (Gd-EOB-DTPA) were not changed at MCD diet for 2 weeks fed mice and were prolonged at MCD diet for 6 weeks fed mice [25]. Therefore, in the evaluation of ^18^F-BMS uptake, there may be little influence of hepatic blood flow. Further study will be needed to clarify the effect of blood flow on ^18^F-BMS uptake in the liver of MCD mice. Thus, the decrease of ^18^F-BMS hepatic uptake might be due to the decrease of MC-1 activity rather than the hepatic perfusion in mice fed a MCD diet for 2 weeks. In our previous study, hepatic clearance of ^99m^Tc-MIBI was changed in mice fed with MCD diet for 2 weeks. These changes have indicated that hepatic mitochondrial membrane potential was decreased at 2 weeks after MCD diet. Thus, non-invasive mitochondrial function imaging such as ^18^F-BMS and ^99m^Tc-MIBI might be useful for NAFLD evaluation.

## Conclusions

Hepatic uptake of ^18^F-BMS was decreased early in mice fed a MCD diet and correlated with hepatic MC-1 activity. This study indicated that ^18^F-BMS PET imaging might be useful for evaluating mitochondrial dysfunction in the early phase of NAFLD in patients.
